# Proceedings of Rochester Hahnemannian Society

**Published:** 1888-07

**Authors:** W. H. Baker

**Affiliations:** Secretary


### PROCEEDINGS OF THE ROCHESTER HAHNEMANNIAN SOCIETY.

The regular monthly meeting of the Rochester Hahnemannian Society was called to order at the office of Dr. J. A. Biegler, April 17th, by the President, Dr. Grant.

Members present: Drs. Grant, Biegler, Schmitt, Carr, Hoard, Hermance, and Baker.

Minutes were read and approved.

After the regular business was finished the Secretary read sections 116 to 131 from the *Organon*, which were the directions given by Hahnemann for proving of drugs, with the following discussion:

Dr. Schmitt—In considering the instructions given by the sections read, the late provings, excepting a few, are of little use. Take the provings by that "*grand society*," the American Institute; they are of no use. The old, reliable drugs, given by the older members of our school, are as reliable to-day as ever.

Provings of Cauloph. and even Berberis are not as reliable as the provings by those old fellows. The great difficulty is getting one in the condition to prove, as Hahnemann teaches. If we could get the inmates of a monastery to help us we might make more progress.

Dr. Biegler—The only way I have to notice the effect of medicines is in the sick as I prescribe. I cannot give up and follow the instructions given in sections read.

I know of one or two persons that stand ready to prove as I direct, but they are not reliable.

Many speak to me of the effect of the first powder I give. I saw a lady a few days ago in the street who came to me nearly blind. As I was passing her, thinking she did not know me, she hailed me, and told me the first powder I gave her and another to be taken some days after were the powders that did her the most good. She was taking Sacch. lact. between. She knew by the effect which powders were medicine. I smiled, thinking she did not see me, but she did, as her sight was much better.

Dr. A. C. Hermance then read a paper on constipation and treatment, and discussion followed.

Dr. Biegler—I think this a good paper and one that may do much good, as it may fall in the hands of some that want light and be a great help. I think it a better paper than any read before the American Institute last year.

Dr. Schmitt—I think this a practical paper, and enough of it to help us in our every-day practice. When I first began the practice of Homœopathy, I used to carry with me a paper written by Dr. Ad. Lippe on cholera infantum. That helped me out many times. We ought to have more of these papers, for they do much good.

Moved and seconded the paper be accepted and published in THE HOMŒOPATHIC PHYSICIAN. Carried.

Dr. Carr was appointed essayist for next meeting.

Adjourned to Dr. Schmitt's office in one month.

---

May 15th, 1888, the regular monthly meeting of the Rochester Hahnemannian Society was called to order at the office of Dr. Julius Schmitt by the President, Dr. R. C. Grant.

Members present: Drs. Grant, Biegler, A. C. Hermance, Schmitt, Hoard, and Baker. Dr. Brownell, of this city, and Dr. Johnson, of Pittsford, N. Y., were present as visitors.

Minutes were read and approved.

Sections 131 to 145 inclusive were read from the *Organon*, which was the continuation of the investigation of remedies as to the proving of them upon the healthy and use in the sick.

Discussion followed.

Dr. Baker—One point I notice is that Hahnemann advises one dose. If we wish to get the pure effect of the drug, and allow it to act the same as when we prescribe for the sick, to give a dose and then let it alone.

Dr. Grant—How wonderfully explicit he is in every detail!

Dr. Schmitt—I will illustrate by reciting a case. A short time ago a messenger came to my office to get medicine for another who had colic, as it had been relieved before by Sulph. I sent that remedy, which gave no relief. I saw the patient the next day and found the following conditions: *Pain in the right side, extending up to the hypocondrium and to back; pain shooting up from abdomen into chest on both sides; better lying on the painful side;* worse about three P. M. Lycopod.$^{mm}$ cured. I gave the above remedy because I remembered much the same condition in myself, which proves we remember symptoms when experienced by ourselves better than when we read them. I had the pain in my right side, for which I could not account, unless it was caused by part of a toothpick I had swallowed, and which gave me some anxiety. As I was lying in an easy chair I found myself moving my tongue back and forth. I immediately jumped up and took a dose of Lyc. I slept all that night, and was well the next day.

Dr. Carr, the essayist of the evening, being absent from illness, Dr. Schmitt proposed the "Class Talks," by Professor Kent, published in the last number of HOMŒOPATHIC PHYSICIAN, be read, as they might interest those present. He said papers like that lift a man right up. After the reading of "Class Talks" the following discussion took place:

Dr. Brownell—I would like to hear your idea, Dr. Biegler, in regard to the treatment of tape-worm, according to the article just read.

Dr. Biegler—He is right. I believe there are always enough symptoms in each case to make a prescription, and it is the only way to *cure* them. We must prescribe for the patient and not the worm. Some time ago a boy was brought to me for treatment. His father had had a tape-worm, and finally expelled it with pumpkin-seed tea. The son had a worm, and for over three years before he came to me had taken pumpkin-seed tea without relief.

Under treatment the worm was finally expelled, but the boy did not get well. Marked symptoms for Gamboge came up, which cured all the symptoms and the boy remained well.

I reported the case at the last meeting of the I. H. A., and it was published in THE HOMŒOPATHIC PHYSICIAN, Nov., 1887, page 428.

Another case is a gentleman. I gave Felix mas $\phi$, together with the anti-psoric remedy. The worm was expelled, but the patient did not regain his health and is still under treatment. This treatment is recommended by Hahnemann.

Dr. Brownell—I prescribed pumpkin-seed tea in the case of a boy with a tape-worm, who had ceased to grow, was puny and in bad health. The worm was soon expelled, and the effect was wonderful. He began to grow and get fat, and in six months you would not have known him, and he remains in the best of health.

Dr. Schmitt—It may be pumpkin-seed tea was homœopathic, for it will not expel the worm in every case.

So far I have been successful in only one case. The patient had never been well, and after he was rid of the worm did not regain his health. This case proves that simply the expulsion of the worm will not cure the case. June 21st, 1883, he received a dose of Natr. carb.$^{cm}$, which was soon followed by pieces of tape-worm.

July 5th, another dose of 30 M. Between this dose and the next he received Berberis for a urinary trouble, which only palliated the following symptom, for which it was given—cutting colic, with nausea.

August 5th he came to me with a worm's head.

September 10th he came with another piece of worm. He received another dose of Natr. carb.$^{cm}$, and finally there was a second head expelled. There were, no doubt, two worms.

From September until January he had no medicine. He then received Sulph., followed with Merc., the trouble now being a calculus. Indications came up for Mezer. *At stool he had tenesmus of the bladder, and a stitching pain in the penis.* In the evening he had to urinate, during which he felt something pass, and on examination found a calculus. The point I wish to make is, the patient does not get well after simply expelling the worm.

Dr. Biegler—Calculi can be expelled with our remedies if the calibre of the urethra will allow.

Dr. Hoard was appointed essayist.

Adjourned to Dr. Biegler's office, the second Tuesday in June.

DR. W. H. BAKER, *Secretary.*

